# Pharmacological Effects of Ginseng on Liver Functions and Diseases: A Minireview

**DOI:** 10.1155/2012/173297

**Published:** 2012-09-11

**Authors:** Nguyen Huu Tung, Takuhiro Uto, Osamu Morinaga, Young Ho Kim, Yukihiro Shoyama

**Affiliations:** ^1^Faculty of Pharmaceutical Sciences, Nagasaki International University, 2825-7 Huis Ten Bosch, Sasebo, Nagasaki 859-3298, Japan; ^2^College of Pharmacy, Chungnam National University, Daejeon 305-764, Republic of Korea

## Abstract

Ginseng, an ancient and famous medicinal herb in the Orient, has been used as a valuable tonic and for the treatment of various diseases including hepatic disorders. Ginseng saponins, commonly known as ginsenosides, are principal constituents and have believed to be responsible for multiple ginseng health benefits. There are more 40 ginsenosides isolated from ginseng. To date, treatment options for common liver diseases such as cirrhosis, fatty liver, and chronic hepatitis remain problematic. In this regard, ginseng extracts and individual ginsenosides have shown a wide array of beneficial role in the regulation of regular liver functions and the treatment of liver disorders of acute/chronic hepatotoxicity, hepatitis, hepatic fibrosis/cirrhosis, hepatocellular carcinoma, and so on in various pathways and mechanisms. In this paper, we first outline the pharmacological effects of ginseng and ginsenosides on the liver functions.

## 1. Introduction

Botanical medicines have been applied for the treatment of various human diseases with thousands of years of history in Asia and are sharing a large market in the form of drugs, dietary supplements, and foods. In the west, botanical medicines are categorized as complementary/alternative medicines, dietary supplements, or foods. Ginseng, referred to as the root of *Panax ginseng* C.A. Meyer (Araliaceae), is one of the most valuable medicinal plants, particularly in Korea, China, and Japan [1].

**Figure 1:**
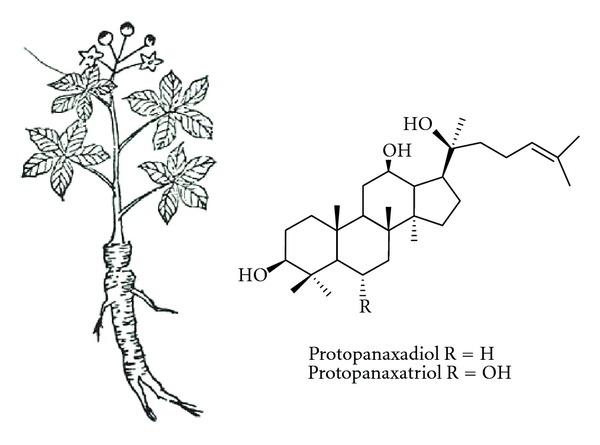
*Panax ginseng* C.A. Mayer and ginsenoisde skeletons.

Ginseng has been used as a valuable tonic and for the treatment of various diseases [1, 2]. Traditionally, ginseng has been processed to make white ginseng (air-drying after harvest) and red ginseng (steaming or heat process) to enhance its preservation and efficacy. In which, red ginseng is more common as an herbal medicine than white ginseng because steaming induces changes in the chemical constituents and enhances the biological activities of ginseng [1, 2]. The pharmacological properties of ginseng are mainly attributed to ginseng saponins, commonly called ginsenoisdes, the major and bioactive constituents [3, 4]. With the development of modern chromatography, there are more 40 ginsenoisdes such as ginsenoisdes Rb_1_, Rb_2_, Rg_1_, Rd, and Re identified from ginseng up to date [4, 5]. Except for ginsenoisde Ro and polyacetylene ginsenoisde Ro belonging to oleanane-type saponins, other ginsenoisdes are of dammarane-type saponins and classified into protopanaxadiol and protopanaxatriol groups depending on whether or not hydroxyl group at C-6 of aglycon moieties exist (Figure 1). On the other hand, ginseng and ginsenoisdes have been found to exhibit multiple pharmacological activities via different mechanisms and pathways *in vitro*, *in vivo*, and clinical models [2–4]. Having been well documented, there are hundreds of research papers as well as extensive reviews spotlighted on individual topics, that is, cardiovascular [6, 7], central nervous [8], and immune systems [9, 10]. However, the pharmacological effects of ginseng/ginsenoisdes on liver disorders have not been systematically reviewed. The purpose of this paper is to introduce the multifaceted pharmacological effects and related mechanisms of ginseng/ginsenoisdes on hepatic functions.

## 2. Liver Diseases and Herbal Medicine in the Treatment of Liver Diseases

Liver diseases represent a major health burden worldwide, with liver cirrhosis being the ninth leading cause of death in western countries [11]. Chronic viral hepatitis B and C, alcoholic liver disease, nonalcoholic fatty liver disease, and hepatocellular carcinoma are the major entities and many problems remain unresolved. Therapies developed along the principles of western medicine are often limited in efficacy, carry the risk of adverse effects, and are often too costly, especially for the developing world. Therefore, treating liver diseases with plant-derived products which are accessible and do not require laborious pharmaceutical synthesis seems highly attractive. Furthermore, in spite of the advances in conventional medicine in the last decades, professionals and the lay public of developed countries pay increasing attention to phytomedicine. Several recent surveys from Europe and the United States have demonstrated a sharp rise in the use of botanical drugs within a few years, and up to 65% of patients with liver disease take herbal preparations [12–15]. 

## 3. Protective Effect of Ginseng Extracts and Ginsenoisdes on Hepatic Functions and Diseases

### 3.1. Ginseng Extracts

#### 3.1.1. Multiple Efficacies

**General Hepatotoxicity**

Acute/chronic hepatotoxicity suffers from drugs used, alcohol, contaminant, and poisoning and may be incidence of liver diseases including liver hepatitis, cirrhosis/fibrosis, and hepatocellular carcinoma (HCC). Ginseng extracts have been reported to show protective effects on hepatocytes *in vitro* and liver injury in various animal, and clinical models induced by a wide variety of hepatotoxins including hydrogen peroxide (H_2_O_2_) [16], alcohol [17, 18], carbon tetrachloride (CCl_4_) [16, 19–22], aflatoxin B_1_ [23, 24], fumonisins [24], tert-butylhydroperoxide (t-BHP) [25], cadmium chloride (CdCl_2_) [26, 27], benzo[alpha]pyrene (BP) [28], 2,3,7,8-tetrachlorodibenzo-*p*-dioxin [29], lipopolysaccharide [30], diethylnitrosamine [31], galactosamine (GalN) [32–34], bromobenzene [35], xenobiotics [36], and mercuric chloride [37]. Ginseng has also found to protect liver cells from radiation [38] and viral hepatitis [39].

**Liver Regeneration and Transplantation**

Ginseng is beneficial in the treatment of acute graft-versus-host disease, a rare complication after liver transplantation with an extremely poor prognosis [40]. On the other hand, red ginseng is markedly effective in liver regeneration after partial hepatectomy in rats (70%) [41] and dogs (40%) [42] by increase of the liver weight and acceleration of hepatocyte proliferation.

**Fatty Liver and Liver Glycogen**

Liver and adipose tissue play an important role in both lipid and sugar metabolism. It has been found that ginseng has effects on lipid and sugar when administrated to rats *in vivo* [43, 44]. Hepatic cholesterol and triglyceride contents are decreased and phospholipid is increased by ginseng administration in the high cholesterol diet-fed rats, supporting to improvement of the fatty liver [45]. In another study, ginseng produces a decrease of hepatic glycogen content in fat diet-fed rats [44].

**Liver Cancer**

HCC is one of the most frequent tumor types worldwide. It is the fifth most common cancer and the third leading cause of cancer death [46]. Ginseng has been evaluated as a chemopreventive therapy against HCC [47–49]. Results of epidemiological studies of ginseng intake and cancer cases (4600 patients) show that those who take ginseng are less likely to contract various cancers such as cancer of the stomach, liver, and lung than those who do not take it, and that increased intake leads to a lesser ratio of danger, proving its usefulness for primary prevention [50, 51]. In a clinical survey, the prolonged administration of red ginseng extract significantly inhibits the incidence of hepatoma and also proliferation of pulmonary tumors induced by aflatoxin B_1_ and urethane [52]. Recently, another clinical research reported that ginseng administration induced a significant improvement in liver function tests, decreased the tumor marker levels, and decreased the viral titers in HCV (hepatitis C virus) patients, supporting powerful therapeutic effects against HCV and liver cancer [39].

#### 3.1.2. Mechanisms of Actions

**Antioxidation**

The mechanisms which provide ginseng's hepatoprotective effects are closely attributed to antioxidation properties. Ginseng enhanced the antioxidant defense mechanism [53] and increased self-antioxidant enzyme activities of superoxide dismutase (SOD), catalase (CAT), glutathione peroxidase (GPx), glutathione reductase (GR), glutathione-S-transferase (GSH), and heme oxygenase-1 in the aged-rat liver [54] and hepatotoxins-induced liver damages in rats [20, 23, 26, 28, 30, 55, 56]. Ginseng treatments inhibited oxidative stress damage such as lipid peroxidation [16, 26, 28, 54], malondialdehyde [23], thiobarbituric acid reactive substance [23, 30, 56], alanine aminotransferase (ALT), aspartate aminotransferase (AST), and lactate dehydrogenase (LDH) [25, 28, 39, 42, 57]. The protective effects have been histologically and histochemically monitored. Recently, further molecular mechanism studies found that ginseng suppresses mitogen-activated protein kinase (MAPK) signals [16], nuclear factor-kappa B (NF-kB), and inducible nitric oxide synthase (iNOS) protein expression [30, 56].

**Anti-Inflammation**

Inflammatory effects of ginseng have been responsible for the liver protection. Ginseng suppressed the production of inflammatory cytokines (IL-1*β*, IFN-*γ*) and chemokines (MCP-1, MIP-2*β*, KC) in CCl_4_-treated mice [55]. Recently, ginseng was found to inhibit tumor necrosis factor alpha (TNF-*α*)-stimulated NF-kB activation and further suppressed the gene expression of iNOS and cyclooxygenase-2 (COX-2) in HepG-2 cells [58].

**Inhibition of Cytochrome P450 (CYP)**

Ginseng and ginsenoisdes may have selectively inhibitory effects on CYP activities. In a recent animal study, ginseng was found to inhibit specifically benzo [alpha]pyrene-induced CYP1A1 activation by downregulation of the gene expression [28]. Another study also indicated that the inhibitory effects of ginseng on CYP enzymes take part in the protection of rat liver microsomes against CCl_4_ [21]. Further investigation into ginseng metabolism suggested that ginsenoisde metabolites like compound K, produced after oral administration, are responsible for the inhibition of the CYP-mediated metabolism rather than naturally occurring ginsenoisdes [59]. These evidence may explain some of the hepatoprotective effects of ginseng against hepatotoxins.

### 3.2. Ginsenoisdes

#### 3.2.1. Ginsenoisde Rb_1_

Ginsenoisde Rb_1_, a major ginseng saponin, protects hepatocytes against t-BHP and CCl_4_ by regulating inducible hepatic enzymes of ALT and AST [22, 60] and may be associated with modulating liver CYP activation [61] and protein phosphorylations [62]. In a recent study, ginsenoisde Rb_1_ significantly inhibits liver fibrosis by inhibiting activation, proliferation, and expression of collagen, transforming growth factor-*β*_1_ (TGF-1), matrix metalloproteinase (MMP)-2, and tissue inhibitor of metalloproteinase (TIMP)-1 in hepatic stellate cells, the major cause of liver fibrosis, at 10–80 *μ*g/mL [63]. More recently, ginsenoisde Rb_1_ is found to inhibit TNF-*α*-mediated NF-kB transcriptional activity in HepG2 cells with IC_50_ of 27.45 *μ*M and gene expression of iNOS and COX-2 inducible inflammatory enzymes [58].

Ginsenoisde Rb_1_ is beneficial against fatty/high cholesterol liver as well as hepatic triglycerides in rats fed on high-fat diet, supporting that ginsenoisde Rb_1_ may be involved in lipid metabolism by regulating the activity of microsomal CYP monooxygenase and 3-hydroxy-3-methyl glutaryl coenzyme A (HMG-CoA) reductase [64–66].

#### 3.2.2. Ginsenoisde Rg_1_

Ginsenoisde Rg_1_, also one of the most abundant ginseng components of ginseng, significantly improves the extent of liver fibrosis in rat induced by thioacetamide as reducing the serum levels of fibrotic markers of ALT, AST, and alkaline phosphatase and hepatic hydroxyproline content [67]. In addition, in cultured hepatic stellate cells, ginsenoisde Rg_1_ markedly inhibits cell proliferation, activation and formation of reactive oxygen species, and the NF-kB activity [67]. Also, ginsenoisde Rg_1_ is found to inhibit TNF-*α*-mediated NF-kB transcriptional activity in HepG2 cells with IC_50_ of 28.14 *μ*M and gene expression of iNOS and COX-2 inducible inflammatory enzymes [58].

Recently, ginsenoisde Rg_1_ suppresses hepatic glucose production via AMP-activated protein kinase (AMPK) activation in HepG2 cells [68].

#### 3.2.3. Ginsenoisdes Rd, Re, and Ro

Yuan et al. reported that ginsenoisde Re (40 mg/kg) attenuates alcoholic fatty liver disease through regulation of AMPK and mitogen-activated protein kinase (MAPK) pathways in alcoholic-fed ICR mice [69]. In another related study, the same group revealed ginsenoisde Re to lower blood glucose and lipid level via activation of AMPK pathway in HepG2 cells and high-fat diet fed mice, suggesting therapeutic benefits on type 2 diabetic patients with insulin resistance and dyslipidemia [70].

Ginsenoisde Rd is recently found to inhibit TNF-*α*-mediated NF-kB transcriptional activity in HepG2 cells with IC_50_ of 12.05 *μ*M and gene expression of iNOS and COX-2 inducible inflammatory enzymes [58].

Ginsenoisde Ro, an oleanane-type ginsenoisde, (50 and 200 mg/kg, p.o.) shows the protective effects on GalN and CCl_4_-induced acute hepatitis rats as monitored by the level of inducible hepatic enzymes of glutamic oxaloacetic transaminase and glutamic pyruvic transaminase [32].

#### 3.2.4. Ginsenoisdes Rg_2_, Rg_3_, and M_1_

Ginsenoisdes Rg_2_ and Rg_3_ represent major components of red ginseng [5]. Ginsenoisde M_1_ or compound K is an intestine bacterial metabolite of the ginseng protopanaxadiol-type saponins. In a recent study, ginsenoisde Rg_2_ significantly inhibits hepatic glucose production in HepG2 cells via activation of AMPK pathway like ginsenoisde Re [71]. Meanwhile, ginsenoisdes Rg_3_ and M_1_ have well evaluated for their cancer chemopreventative against several cancer cell lines and tumors *in vitro* and *in vivo* including leukemia, colon, and HCC [51, 72–74]. More recently, ginsenoisde Rg_3_ at 25–200 *μ*g/mL shows significantly antiproliferative effects on liver cancer cells (SMMC-7721, Hep1-6, and HepG2) and hepatocellular tumor growth *in vivo* by inhibiting cancer cell proliferation and promoting cancer cell apoptosis [75, 76]. In another research, ginsenoisde M_1_ exhibits antihepatocellular carcinoma activity by augmenting apoptosis via Bid-mediated mitochondrial pathways [77]. On the other hand, ginsenoisde M_1_ has beneficial role against hyperlipidemia and obesity by attenuating hepatic lipid accumulation via AMPK activation in human hepatoma cells [78]. In addition, ginsenoisdes Rg_3_ and M_1_ are also involved in general hepatoprotection against hepatotoxins [60, 79].

## 4. Application of Ginseng to Hepatic Diseases

Liver disorders are among the most serious sicknesses in the population. Together with healthy lifestyle recommendation, therapies for hepatic diseases are necessary and need be more effective and economical [80]. Botanical medicines have been used traditionally worldwide for the prevention and treatment of liver disease. By now, herbal products are increasingly consumed, mainly in chronic liver disease. Turmeric (*Curcuma longa*), green tea (*Camellia sinensis*), garlic (*Allium sativa*), licorice (*Glycyrrhiza *spp.), especially milk thistle (*Silybum marianum*) with have been well researched and recognized as phytotherapies for use in a wide variety of prevention and treatment of hepatic diseases [81, 82].

Based on compelling experimental evidence, ginseng has an interesting hepatoprotective profile. From a therapeutic standpoint, ginseng could provide an alternative phytomedicine and another option for the preventative therapy and treatments of common liver diseases such as cirrhosis, fatty liver, and chronic hepatitis in addition to a well-known tonic and in form

s of healthy foods.

## 5. Conclusions

Ginseng and its principal components, ginsenoisdes, have shown a wide array of pharmacological activities including beneficial role in the regulation of liver functions and the treatment of liver disorders of acute/chronic hepatotoxicity, hepatitis, hepatic fibrosis/cirrhosis, liver hepatectomy, liver transplantation, and even liver failure and HCC. The possible activity pathways of the actions have been also investigated. There is increasing attention to the effects of ginseng on the liver functions. However, more detailed molecular mechanisms of the activities of ginseng/ginsenoisdes as well as further efficacy and safety studies remain to be explored.

It is another important area for further research and development to combine ginseng with other liver active drugs to investigate their possible synergic efficacy and preferably pharmacological properties. 

Taken together, accumulating evidence supports the potential of ginseng in the treatment of the hepatic diseases and further studies will facilitate their application so far.
